# Influence of Slow-Lead Poisoning on the Product of Conception

**Published:** 1860-07

**Authors:** 


*Influence of Slow Lead-Poisoning on the Product of Conception.*—M. C. Paul, resident physician to a Paris Hospital, has collected 81 cases showing the baneful influence of lead. His attention was attracted to the subject by the case of a woman who had given birth to three healthy children before she took to the occupation of printing-type cleaning, and who out of ten subsequent gestations had had eight miscarriages.

One child, which was born at full time, died at five months. The 81 cases refer mostly to women, only a few relating to men. From these cases M. Paul thinks himself justified in supposing that lead-poisoning has not only the already well-known effects, but that it also causes the death of the fœtus, or the premature decease of the child, whether either the father or mother has been exposed to lead-poisoning. Out of the 123 gestations included in the 81 cases, 64 abortions, 4 premature labors, and 5 still-births were noted. It was, moreover, observed that 20 children, born at full time, died within their first year, 8 within their second, 7 within their third, and one only at a later period. 14 children are now alive, 10 of whom are above three years old. Metrorrhagia occurred in 15 cases, the loss of blood depending, very probably, on abortions.—*Gazette des Hopitaux.*

